# Category-Specific versus Category-General Semantic Impairment Induced by Transcranial Magnetic Stimulation

**DOI:** 10.1016/j.cub.2010.03.070

**Published:** 2010-05-25

**Authors:** Gorana Pobric, Elizabeth Jefferies, Matthew A. Lambon Ralph

**Affiliations:** 1 Neuroscience and Aphasia Research Unit, School of Psychological Sciences, University of Manchester, Manchester M13 9PL, UK; 2 Department of Psychology, University of York and York Neuroimaging Centre, York Y010 5DD, UK

**Keywords:** SYSNEURO

## Abstract

Semantic cognition permits us to bring meaning to our verbal and nonverbal experiences and to generate context- and time-appropriate behavior [1–2]. It is core to language and nonverbal skilled behaviors and, when impaired after brain damage, it generates significant disability [3]. A fundamental neuroscience question is, therefore, how does the brain code and generate semantic cognition? Historical and some contemporary theories emphasize that conceptualization stems from the joint action of modality-specific association cortices (the “distributed” theory) [4, 5] reflecting our accumulated verbal, motor, and sensory experiences. Parallel studies of semantic dementia, rTMS in normal participants, and neuroimaging indicate that the anterior temporal lobe (ATL) plays a crucial and necessary role in conceptualization by merging experience into an amodal semantic representation [1, 2, 6–8]. Some contemporary computational models suggest that concepts reflect a hub-and-spoke combination of information—modality-specific association areas support sensory, verbal, and motor sources (the spokes) while anterior temporal lobes act as an amodal hub. We demonstrate novel and striking evidence in favor of this hypothesis by applying rTMS to normal participants: ATL stimulation generates a category-general impairment whereas IPL stimulation induces a category-specific deficit for man-made objects, reflecting the coding of praxis in this neural region.

## Highlights

► First observation of category-specific semantic impairment in normal participants ► rTMS stimulation of ATL generates a category-general impairment ► Stimulation of IPL induces a category-specific deficit for praxic man-made objects ► Both the ATL hub and the modality-specific regions contribute to semantic representations

## Results

To date, studies have focused on the contribution of either modality-specific association regions or the ATL to semantic memory. Past studies have relied solely upon the study of neurological patients to test the necessity of these different areas to semantic memory. In this rTMS investigation of picture naming in neurologically normal participants, we tested the differential contribution of both regions for the first time. Like neuropsychological studies, rTMS can be used to test the necessity of regions for cognitive function, yet the stimulated regions are much more specific and their location is under the control of the experimenter. It has the additional, unique benefit that the functional contribution of multiple regions can be successively compared within the same participants.

**Figure 1:**
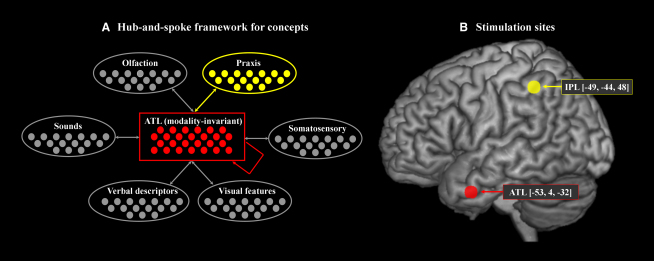
The Hub-and-Spoke Model of Semantic Representation (A) A “hub-and-spoke” computational framework for the generation of concepts. Each oval denotes a different source of information that is represented in modality-specific association cortices. Each of these interacts with an ATL modality-invariant hub which, through the process of translating between all of the motor, verbal, and sensory modalities, generates an additional source of amodal information that codes conceptual rather than surface similarities. (B) Two sites of rTMS (lateral ATL versus left IPL) with the mean MNI coordinates.

We used this approach to delineate between three different hypotheses: (1) distributed-only—concepts reflect the conjoint action of modality-specific areas alone without the ATL; (2) hub-only—in which concepts are formed within the ATL and modality-specific regions only provide sensorimotor input/output gateways rather than making a necessary contribution to conceptualization; (3) hub-and-spoke—in which modality-specific regions provide the basic sensory, motor, and verbal ingredients while the ATL hub supports an additional amodal representation which codes the pan-modal, deep statistical structure and thus generates a high-dimensional, modality-independent similarity matrix [9, 1]. Under this latter account, both the ATL “hub” and modality-specific “spokes” provide necessary and important contributions to conceptualization (see Figure 1). We adjudicated between these competing theories by investigating category-specific impairments. To date, these have only ever been observed clinically; some patients present with relatively greater problems for one domain than another (e.g., poorer performance for animals than man-made items) [10, 11]. Specifically, we compared the effect of stimulating the ATL and IPL in normal participants (by applying rTMS off-line for 10 min at 1 Hz (600 s at 120% motor threshold level) over left ATL (−53, 4, −32), left IPL (−49, −44, 48), and occipital pole (Oz—as a control site) prior to naming pictures and numbers (see Experimental Procedures). If the ATL is involved in semantic memory, as proposed, then rTMS should generate a category-general effect. If the IPL spoke is implicated then stimulation should impact on semantic performance but only for concepts that rely on praxis information—i.e., manipulable man-made objects. Thus stimulation at this site should induce a category-specific impairment which, as far as we are aware, has never been demonstrated before in neurologically intact participants.

### Overall Naming

**Figure 2:**
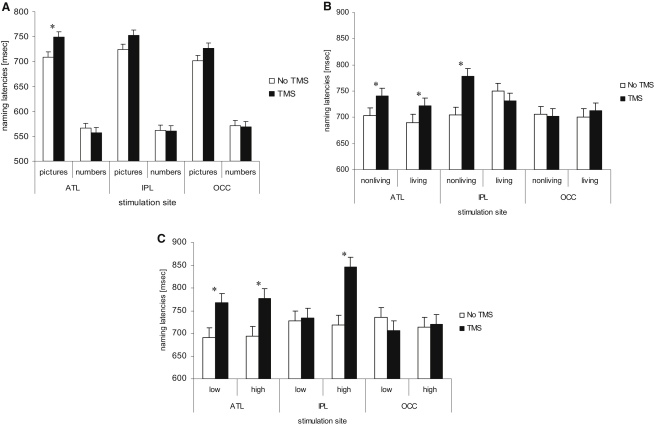
The Effect of rTMS Stimulation on Naming Each panel shows the effect of three different cortical sites of stimulation on naming latencies. (A) Pictures versus numbers. (B) Living versus nonliving items. (C) Low- versus high-manipulable objects. Abbreviations: ATL, anterior temporal lobe stimulation; IPL, inferior parietal lobule; OCC, occipital pole. Asterisk denotes significantly slower naming times after rTMS than at baseline. Note: error bars indicate SEM adjusted to reflect the between-condition variance used in repeated-measure designs [15].

The response times (RT) for all participants and all conditions were submitted to a repeated-measures ANOVA with three within-subjects factors: task (picture naming, number reading), site (left ATL, left IPL, and Oz), and TMS (no-TMS versus rTMS). A main effect of task was observed (F = 73.891, df = 1,8, p < 0.001) as well as a significant interaction between task and TMS (F = 8.402, df = 1,8, p < 0.05). Planned comparisons t tests were used to compare performance for each stimulus set (pictures and numbers) with and without TMS at each site (see Figure 2A). After controlling for false discovery rate (FDR) [12], only the left ATL stimulation significantly slowed performance for the picture naming task [t(8) = 3.3, p < 0.05].

### Error Analyses

The error rate was examined in a repeated-measures ANOVA with stimulus (pictures, numbers), site (left ATL, left IPL, and Oz), and TMS (no-TMS, rTMS) as factors. There were no significant main effects or interactions (p > 0.1). This result is not surprising given that picture naming is a relatively straightforward simple cognitive task. This also held true for all subsequent error analyses (category and manipulability effects).

### Category Effects

From original 200 items, we created two lists of living and nonliving items for category analyses. Final lists contained 35 pairs of items matched for familiarity, frequency, and visual complexity via the *Match* program [13]. The RTs for all participants and all conditions were submitted to a repeated-measures ANOVA with three within-subjects factors: category (living, nonliving), site (left ATL, left IPL, and Oz), and TMS (no-TMS versus rTMS). The main effects were not significant p > 0.08. However, there was a significant three-way interaction between category, site, and TMS (F = 5.39, df = 2,16, p < 0.05). FDR controlled t tests compared performance for each category (living, nonliving) with and without TMS at each site (see Figure 2B). Stimulation of left ATL significantly slowed performance for both living [t(8) = 2.391, p < 0.05] and nonliving [t(8) = 2.394, p < 0.05] items. Left IPL stimulation also reliably slowed responses for nonliving items only [t(8) = 3.1, p < 0.05]. In addition, TMS delivered to the occipital control site had no significant effects on naming living and nonliving items.

### High- versus Low-Manipulability Items

Two new sets of items were selected to explore the impact of rTMS on praxic and nonpraxic items. Specifically, 16 pairs of man-made items were selected that had high- (m = 4.4/5) or low- (m = 2.2/5: from [14]) manipulability ratings. All items were matched pairwise for familiarity, frequency, and visual complexity, using the *Match* program [13]. RTs for all participants and all conditions were submitted to a repeated-measures ANOVA with three within-subjects factors: manipulability (high, low), site (left ATL, left IPL, and Oz), and TMS (no-TMS versus rTMS). A main effect of TMS was observed (F = 6.61, df = 1,8, p < .05) as well as a significant interaction between TMS and site (F = 6.08, df = 1,8, p < 0.05). FDR controlled t tests compared performance for each stimulus set (high versus low manipulable) with and without TMS at each site (see Figure 2C). Stimulation of left ATL significantly slowed performance for both low-manipulable [t(8) = 2.34, p < 0.05] and high-manipulable [t(8) = 2.31, p < 0.05] items. Crucially, left IPL stimulation also reliably slowed responses but for high-manipulable items only [t(8) = 4.21, p < 0.05]. It did not have an effect on the low-manipulable items. In addition, TMS delivered to the occipital control site had no significant effects on naming living and nonliving items.

## Discussion

ATL stimulation generated a selective slowing of basic level naming but had no impact on number naming, adding to previous evidence for the selective involvement of the ATL in semantic processing [6, 16]. Stimulation of the occipital control site had no impact on either task, indicating no generalized, nonspecific effect of rTMS. There was, however, a nonsignificant slowing after IPL stimulation (see Figure 2A). Further analyses showed that this partial effect was due to a category-specific pattern.

This was explored with respect to two matched sets of living and nonliving items (see Figure 2B). Each stimulation site produced significantly different effects on the two categories. There was a category-general effect for ATL stimulation—i.e., slowing the naming of both living and nonliving items. In contrast, left IPL stimulation generated a category-specific effect—slowed responses only for nonliving items. To confirm that the IPL category effect reflected the coding of praxis information, we compared the effect of rTMS to two matched sets of manipulable versus nonmanipulable, man-made items. The same pattern emerged across the three sites: ATL stimulation slowed both sets of man-made items (low- and high-manipulable items). Crucially, left IPL stimulation slowed responses for high-manipulable items only. TMS delivered to the occipital control site had no significant effects on naming times.

Through the use of rTMS in normal participants, we have been able to demonstrate two contrasting effects. Stimulation of the ATL leads to a generalized slowing of semantic processing across all types of concept (living, manipulable objects and nonmanipulable man-made items). This is in keeping with the category-general deficits observed in the context of the ATL-focused atrophy underlying semantic dementia [17]. In contrast, stimulation to the IPL generates a category-specific pattern reflective of the praxis information coded in this neural region. These results are consistent with the category-specific pattern observed in stroke patients with lesions in this same region [18]. As far as we are aware, this is the first time that a category-specific naming deficit has been generated in normal participants.

The findings of this study fit squarely with the hub-and-spoke model of semantic memory and rule out the other models. Both the ATL amodal hub and the modality-specific association “spokes” contribute to semantic representations. Because the ATL hub is involved in the translation and deeper encoding of pan-modal information sources, the representations become modality invariant [1, 2, 9] and thus they are involved in conceptualization for all types of category. In contrast, modality-specific information contributes only to the subset of concepts that are experienced in that modality. Unlike other modality-specific areas, IPL is an ideal test region given that there is an almost binary division of praxis experience between manipulable objects and other concepts [18].

By demonstrating these contrasting region-specific effects within the same participants, this study resolves two key issues about semantic memory. First, the literature has debated two theories of semantic memory: (1) classical models in which concepts are formed by the mass-action of multiple, modality-specific sources of sensory, verbal, and motor information (the distributed-only model) [4, 5] and (2) a view in which the anterior temporal lobes act as a representation hub over which modality-specific sources of information are combined and rerepresented to form concepts (the hub-only model). The results from this study are consistent with elements from both approaches and reject models that posit a sole role for either distributed or hub regions in semantic memory. By demonstrating semantic effects after stimulating either region, we can more firmly conclude that both areas make a critical contribution to conceptualization, in line with the hub-and-spoke approach. Under this account concepts reflect the combination of two sources of information—modality-specific knowledge coded in their respective association cortices and the action of an ATL, rerepresentational hub [1, 2]. Computational models of semantic memory [19] show that it is very difficult, if not impossible, to integrate all disparate sources of modality-specific information successfully via a web-like organization of direct connections. This is because the relationship between individual pieces of information and different concepts is highly complex and nonlinear. In addition, a key characteristic of semantic memory is that it allows us to generalize knowledge on the basis not of superficial but instead of conceptual similarity, and also generalize our previous knowledge to exemplars that we have never experienced before [1, 20]. All these semantic functions can be achieved if modality-specific sources of information are integrated with one or more representational hubs in a hub-and-spoke architecture.

Second, this rTMS study also helps to align contrasting results found in the neuropsychological literature. Research in this area has been driven by two somewhat unconnected sets of patients. On the one hand, there are the striking reports of patients with category-specific impairment. These data suggest that neural systems are organized categorically or, more likely, reflect the fact that concepts from different categories have divergent sources of sensorimotor and verbal information (the differential-weighting hypothesis) [10, 21]. On the other hand, there is a somewhat separate literature on semantic dementia patients. These patients demonstrate that it is possible for brain damage to generate a highly selective yet general impairment of conceptual knowledge [1, 2, 22, 23]. The current rTMS study shows that the contrast between patients with category-specific or category-general semantic impairment are not due to differential material selection across investigators or complex compensatory processes after brain damage. Instead we have been able to demonstrate both types of impairment within the same brain, depending on which neural region is stimulated. We should note here that HSVE patients with a category-specific impairment for living things (the opposite of the category effect induced by IPL stimulation in this study) have ATL damage. In comparison to the inferolateral focus atrophy in SD patients, HSVE patients have significantly greater damage affecting medial temporal regions [17, 24]. Our TMS stimulation was applied to the lateral ATL and this is a much closer match to SD than HSVE.

In conclusion, the hub-and-spoke theory provides a unifying framework for patient and rTMS results. After the original differential-weighting hypothesis, if specific sources of sensorimotor information are suppressed or damaged (e.g., the praxis information coded in IPL regions), then concepts that rely upon this information center are affected. If this source of information is differentially distributed across categories, then when it is suppressed or damaged, a category difference emerges (e.g., performance on praxis man-made items is compromised). In contrast, because the inferolateral ATL hub supports modality-invariant representations [1, 25], then when it is suppressed or damaged, a category-general effect emerges.

## Experimental Procedures

### Design

A 3 × 2 × 2 repeated-measures design was used, with site (left ATL versus left IPL versus occipital pole), task (picture naming versus number reading), and TMS (no TMS versus rTMS stimulation) as the three within-participant factors. The study utilized rTMS with the “virtual lesion” method in which the train of rTMS is delivered offline (without a concurrent behavioral task) and then behavioral performance is probed during the temporary refractory period and compared to performance on the same task outside this refractory window.

### Participants

Nine right-handed participants took part in the experiment (four females; mean age = 20.2 years, SD = 2.1). All participants provided a written consent for participation after being screened for adverse effects of TMS. The experiment was approved by the local ethics committee.

### Stimuli

A total of 200 picture stimuli (from [25]) and 100 number stimuli were used in the basic naming task.

### Task and Procedure

A PC running E-Prime software (Psychology Software Tools Inc., Pittsburgh, PA) allowed the presentation of stimuli and recording of the responses. In a single experimental session, participants named pictures from the AoA battery and read six digit numbers. The experiment began with a practice block of 10 trials for each stimulus set. Experimental trials were presented in a random order in 2 blocks (100 pictures and 50 numbers). After 10 min of offline rTMS, another 2 blocks (100 pictures and 50 numbers) followed. This yielded 300 trials per experimental session. The blocks were randomized across participants. Stimuli were presented until the response was given and followed by a blank screen (duration 500 ms). Verbal responses were recorded with a microphone that was placed in front of each participant. Response latencies were recorded by the computer and the accuracy checked off-line.

### TMS

A MagStim Rapid2 (Magstim Co., Whitland, UK) stimulator with two external boosters was used (maximum output approx. 2.2 Tesla). Magnetic stimulation was applied with a 70 mm figure-of-eight coil.

### Selection of TMS Site

The structural T1-weighted MRI scans were coregistered with the participant's scalp via MRIreg (http://www.mricro.com/mrireg.html). Immediately prior to the TMS session, scalp coordinates were measured with an Ascension Minibird (http://www.ascension-tech.com) magnetic tracking system. The left MNI coordinates for the ATL in standard space were (−53, 4, −32). The coordinates for left inferior parietal lobule (−49, −44, 48) were taken from imaging literature on action and tool semantics [26, 14]. A middle occipital stimulation site (Oz) was also employed as a site to control for possible nonspecific TMS effects.

### Stimulation Parameters

Individual active motor threshold (MT) was determined for every participant. rTMS was delivered off-line for 10 min at 1 Hz (600 s at 120% MT level) applied to the left ATL, left IPL, and Oz. The coil was securely held by experimenter, centered over the site to be stimulated. The average stimulation intensity during rTMS was 63%.

## Supplemental Information

Document S1. Supplemental Experimental Procedures
