# Supplementary material for: Category-Specific versus Category-General Semantic Impairment Induced by Transcranial Magnetic Stimulation
Source: Curr Biol. 2010 May 25;20(10):964–8. doi: 10.1016/j.cub.2010.03.070 (PMC2878637; doi:10.1016/j.cub.2010.03.070)
Supplement: Document S1. Supplemental Experimental Procedures [file mmc1.pdf]

Current Biology, Volume *20*

Supplemental Information

Category-Specific versus Category-General Semantic Impairment Induced by Transcranial Magnetic Stimulation

Gorana Pobric, Elizabeth Jefferies, and Matthew A. Lambon Ralph

Supplemental Information

Supplemental Experimental Procedures

Design – A 3 × 2 × 2 repeated measures design was used, with site (left temporal pole vs. left inferior parietal lobule vs. occipital pole), task (picture naming vs. number reading) and TMS (No TMS vs. rTMS stimulation) as the 3 within-participant factors. The study utilised rTMS using the "virtual lesion" method in which the train of rTMS is delivered offline (without a concurrent behavioural task) and then behavioural performance is probed during the temporary refractory period and compared to performance on the same task outside this refractory window.

Participants – Nine, right-handed participants took part in the experiment (4 females; mean age = 20.2 years, SD = 2.1). All were native English speakers and strongly right-handed, yielding a laterality quotient of at least +90 on the Edinburgh Handedness Inventory [1]. They were free from any history of neurological disease or mental illness and not on any medication. All had normal or corrected-to-normal vision. All gave written informed consent and the experiment was reviewed and approved by the local ethics board. Participants were reimbursed for their participation.

Stimuli –A total of 200 picture stimuli and 100 number stimuli were used in the basic naming task. 200 pictures were taken from an age-of-acquisition (AoA) normed battery [2]. From original 200 items we created two lists of living and non-living items for category analyses. Final lists contained 35 pairs of items matched for familiarity, frequency and visual complexity using the *Match* program [3]. The mean familiarity rating for non-living set was 2.86; SD=0.83 (range from 1.59-4.5); while the mean familiarity rating for the living category was 2.85; SD=0.82 (range from 1.55-4.5). Two new sets of items were selected to explore the impact of rTMS on praxic and nonpraxic items. Specifically, 16 pairs of manmade items were selected that had high (m=4.4/5) or low manipulability ratings

(m=2.2/5) on the ratings from [4]. All items were matched pairwise for familiarity (m=3.02/3.22), frequency (m=42.25/46.19) and visual complexity (m=2.73/2.99), using the *Match* program [3].

Task and procedure - A PC running E-Prime software (Psychology Software Tools Inc., Pittsburgh, USA) allowed the presentation of stimuli and recording of the responses. The participants sat 57 cm in front of a 15” monitor.

In a single experimental session participants were naming pictures from the AoA battery and reading six digit numbers (i.e., 316,565 – “three hundred and sixteen thousand five hundred and sixty five”). This order was counterbalanced across stimulation sites. The experiment began with a practice block of 10 trials for each stimulus set. Experimental trials were presented in a random order in 2 blocks (100 pictures and 50 numbers). After 10 min of offline rTMS, another 2 blocks (100 pictures and 50 numbers) followed. This yielded 300 trials per experimental session. The blocks were randomised across participants. Stimuli were presented until the response was given and followed by a blank screen interval of 500ms. Verbal responses of individuals were recorded using a microphone that was placed in front of each participant. The latency of each response was recorded by the computer and the accuracy checked off-line by listening to the recordings.

TMS - A MagStim Rapid2 (Magstim Co., Whitland, UK) stimulator with 2 external boosters was used (maximum output approx. 2.2 Tesla). Magnetic stimulation was applied using a 70-mm figure-of-eight coil. The double wire windings which make up the figure-of-eight coil carry two alternating electrical currents which converge at the point where the two coils meet (at the centre of the figure-of-eight). A quite focal electrical current can then be induced in the cortex via magnetic conduction from this central point which undergoes minimal attenuation by the intervening soft tissue and bone [5].

Anatomical MRI acquisition - 3D high resolution T1-weighted anatomical images were acquired for all participants using a 3T Philips MR Achieva scanner (Philips Electronics, The Netherlands). MRI scanning parameters included an in-plane resolution of 1 mm and a slice thickness of 1.8 mm with an acquisition matrix of 256 x 256 voxels. The number of contiguous axial slices acquired varied (max = 240) depending on the size of the subject’s head, as full head coverage was required for accurate co-registration of the image to the participants scalp. These high-resolution T1- weighted images enabled reconstruction of the fine individual cortex folding which was used as anatomical landmarks for the TMS targets.

Selection of TMS site – The structural T1-weighted MRI scans were co-registered with the participant's scalp using MRIreg (www.mricro.com/mrireg.html). Immediately prior to the TMS session, scalp coordinates were measured using an Ascension Minibird (www.ascension-tech.com) magnetic tracking system. A series of scalp landmarks were identified for co-registration within the MRI and Minibird coordinates. Once this calibration was complete, the two frames of reference were co-registered using least squares linear estimation. This allowed us to compare the position of the Minibird on the scalp to the underlying cortical surface. From the tip of the temporal pole we measured 10mm posterior along the middle temporal gyrus. This point was used in each participant as an anatomical landmark for the temporal pole (TP). The location of the TP was identified on each participant and the scalp location directly above this site was marked with a permanent marker. The left MNI coordinates for the TP in standard space were (-53, 4, -32). The coordinates for left inferior parietal lobule (-49, -44, 48) were taken from imaging literature on action and tool semantics [6, 4]. A middle occipital stimulation site (occipital pole) was also employed as a site to control for possible non-specific visual effects and also for general arousal effects of TMS induced by somatosensory and acoustic artifacts. According to the international 10-20 electrode system, this site corresponds to the Oz location.

Stimulation parameters - Individual motor threshold (MT) was determined for every participant. Motor threshold was defined as a minimal intensity of stimulation capable of inducing motor evoked potentials in the contralateral FDI muscle greater than 50μV peak-to-peak amplitude in at least 6 out of 10 trials [7] at the optimal scalp position. Repetitive pulse TMS (rTMS) was delivered off-line for 10 min at 1-Hz (600 s at 120% motor threshold level) applied to the left ATL, left IPL, and occipital pole. The coil was securely held by experimenter, centred over the site to be stimulated. This TMS protocol has been shown to produce behavioural effects that last for several minutes after stimulation [8]. For the occipital stimulation the maximal induced current flowed downward with the coil handle pointing upwards. The average MT was 52% of the maximal stimulator output and the average stimulation intensity during rTMS was 63%.

Methodological considerations - An advantage of low frequency rTMS is that rTMS modulates the level of excitability of a given cortical area beyond the duration of the rTMS train itself [8, 9]. In the present design, behaviour was evaluated before and after rTMS. Therefore, a nonspecific disruption of performance due to discomfort, noise, muscle twitches and intersensory facilitation associated with rTMS during the task was avoided. Particular care was taken in the placing of the TP coil because

TMS here is more uncomfortable than over occipital or parietal areas. We manipulated coil orientation (a major factor in the nature of the contraction of facial/neck muscles) to find an orientation that minimized the discomfort to a subjective equivalent to that of the stimulation over other sites. As detailed above, we also used a pattern recognition task as a control to ensure that neither non-specific effects of the rTMS procedure nor task difficulty could explain the observed results.

Supplemental References

1. Oldfield, R.C. (1971). The assessment and analysis of handedness: the Edinburgh inventory. Neuropsychologia. *9*, 97-113.

2. Morrison, C.M., Chappell, T.D., and Ellis, A.W. (1997). Age of acquisition norms for a large set of object names and their relation to adult estimates and other variables. Q.J. Exp. Psychol., *50*, 528-559.

3. Casteren, M. and Davis, M. (2007). Match, A program to assist in matching the conditions of factorial experiments, Behav. Res. Methods *39*, 973-978.

4. Kellenbach, M., Brett, M., and Patterson, K. (2003). Action speak louder than functions. The importance of manipulability and action in tool representation. J. Cogn. Neurosci. *15*, 30–46.

5. Jalinous, R. (1995). Guide to Magnetic Stimulation (Whitland, Wales, Magstim).

6. Noppeney, U., Price, C.J., Penny, W.D., and Friston, K.J. (2006). Two distinct neural mechanisms for category-selective responses. Cereb. Cortex *16*, 437–445.

7. Rossini, P. M., Barker, A. T., Berardelli, A., Caramia, M. D., Caruso, G., Cracco, R. Q., Dimitrijevic, M. R., Hallett, M., Katayama, Y., Lucking, C. H., and et al. (1994). Non-invasive electrical and magnetic stimulation of the brain, spinal cord and roots: basic principles and procedures for routine clinical application. Report of an IFCN committee. Electroencephalogr. Clin. Neurophysiol. *91*, 79-92.

8. Pascual-Leone, A., Tormos, J.M., Keenan, J., Tarazona, F., Canete, C., and Catala, M.D. (1998). Study and modulation of human cortical excitability with transcranial magnetic stimulation. J. Clin. Neurophysiol.*15*, 333-343.

9. Knecht, S., Floel, A., Drager, B., Breitenstein, C., Sommer, J., Henningsen H, et al. (2002). Degree of language lateralization determines susceptibility to unilateral brain lesions.Nat. Neurosci. *5*, 695-699.
